# Prevalence of arboviruses and other infectious causes of skin rash in patients treated at a tertiary health unit in the Brazilian Amazon

**DOI:** 10.1371/journal.pntd.0010727

**Published:** 2022-10-13

**Authors:** Luiz Henrique Gonçalves Maciel, Cosmo Vieira da Rocha Neto, Yasmin Ferreira Martins, Francielen de Azevedo Furtado, Pâmela Cunha Teixeira, Maianne Yasmin Oliveira Dias, Yanka Karolinna Batista Rodrigues, Isa Cristina Ribeiro Piauilino, Sérgio Damasceno Pinto, Aline Cristiane Côrte Alencar, João Bosco de Lima Gimaque, Maria Paula Gomes Mourão, Marcus Vinicius Guimarães Lacerda, Márcia da Costa Castilho, Camila Bôtto-Menezes

**Affiliations:** 1 Programa de Pós-graduação em Medicina Tropical, Universidade do Estado do Amazonas (UEA), Manaus, Amazonas, Brazil; 2 Programa de Iniciação Científica, Fundação de Medicina Tropical Doutor Heitor Viera Dourado (FMT-HVD), Manaus, Amazonas, Brasil; 3 Fundação de Medicina Tropical Doutor Heitor Viera Dourado (FMT-HVD), Manaus, Amazonas, Brazil; 4 Escola Superior de Ciências da Saúde, Universidade do Estado do Amazonas (UEA), Manaus, Amazonas, Brazil; 5 Instituto Leônidas e Maria Deane (ILMD–Fiocruz Amazônia), Manaus, Amazonas, Brazil; Beijing Children’s Hospital Capital Medical University, CHINA

## Abstract

**Background:**

In the clinical course of diseases such as arboviruses, skin rashes may appear, as is often seen in other infectious diseases. The aim of this study was to estimate the prevalence of arboviruses and other infectious causes of skin rash in a tertiary health unit in Manaus, Amazonas state, Western Brazilian Amazon

**Methodology/Principal findings:**

This was a cross-sectional study of patients presenting with rash who sought care at *Fundação de Medicina Tropical Dr*. *Heitor Vieira Dourado* (FMT-HVD) from February 2018 to May 2019. Individuals of either gender, aged over 18 years, were invited to participate voluntarily. Infection by Zika virus (ZIKV), dengue virus (DENV), chikungunya virus (CHIKV), Mayaro virus (MAYV), Oropouche virus (OROV) and measles was evaluated using RT-qPCR (real-time polymerase chain reaction). Immunodiagnostic tests for EBV, CMV, HIV, syphilis, rubella and measles were also performed. A total of 340 participants were included, most were female (228, 67.1%) with an average age of 36.5 years (SD ± 12.2 years). The highest prevalence was of ZIKV monoinfections (65.3%, 222/340), followed by DENV (0.9%, 3/340) and CHIKV infection (0.3%, 1/340). No cases of MAYV, OROV or rubella were found. Other causes of skin rash were detected: measles (2.9%, 10/340), parvovirus B19 (0.9% 3/340), HIV (0.3%, 1/340) and syphilis 0.6% (2/340). The co-infections identified were ZIKV+HIV (0.3%, 1/340), ZIKV+measles (0.3%, 1/340), ZIKV+parvovirus B19 (0.3%, 1/340), ZIKV+EBV (0.3%, 1/340), EBV+parvovirus B19 (0.3%, 1/340), CMV+parvovirus B19 (0.6%, 2/340), CMV+syphilis (0.3%, 1/340), ZIKV+EBV+parvovirus B19 (0.3%, 1/340) and CMV+EBV+parvovirus B19 (0.9%, 3/340). Approximately one quarter of patients had no defined cause for their skin rash (25.3%, 86/340).

**Conclusions:**

Despite the benign clinical evolution of most of the diseases diagnosed in this series of cases, syndromic surveillance of diseases such as syphilis and HIV are of utmost importance. Periodic serosurveillance might also aid in evaluating the trends of endemic diseases and eventual outbreaks.

## Author summary

Arboviruses are transmitted to humans by infected arthropods mostly mosquitoes, many of which lay their eggs in containers with standing water in our homes. Dengue and Zika are some of the examples of these viruses that are responsible for affecting many people in big cities every year. The illnesses can cause fever, malaise, and red patches on the skin that may or may not itch. This study aimed to investigate whether people with red patches on their skin had these diseases transmitted by mosquitoes, in Manaus, Brazil. In addition, we investigated whether they were ill from other causes, as several other illnesses can also cause red patches on the skin. The exams were carried out on 340 people, men and women over 18 years of age. It was identified that 231 people were sick from arboviruses, 227 of which were sick from Zika, 3 from dengue and 1 from chikungunya. Other causes of red spots were also identified, as was the case of sexually transmitted infections syphilis and HIV, with two and three positive cases for each disease, respectively. In addition, some cases of diseases such and measles, which are transmitted by sneezing or coughing, have been identified.

## Introduction

Arboviruses are a group of viruses that are transmitted by arthropods. Most arboviruses belong to the genera *Alphavirus* (*Togaviridae* family) and *Flavivirus* (*Flaviviridae* family); other members that can affect human health are from the *Bunyaviridae*, *Reoviridae*, and *Rhabdoviridae* families [1]. These characteristically have a wide geographic distribution, which is associated with the presence of the vector, and cause asymptomatic infections or febrile diseases in humans in both enzootic and urban cycles [2].

Arboviruses are responsible for over 40,000 deaths per year worldwide and a high burden and cost to health systems [3,4]. Cases of dengue virus (DENV) infection in Latin America, for example, are responsible for spending that exceeds $3 billion annually [5]. In Brazil, for instance, total costs with the management of the principal arboviruses (Zika, dengue and chikungunya) caused an expenditure of US$ 430,711,610 in 2016 [6].

In addition to the economic impacts, some arboviruses interfere directly with the patient’s occupational life; for example, although most CHIKV-infected patients show full recovery after the acute phase of the disease, severe arthralgia can last weeks or months, whereas ZIKV is capable of developing a syndrome called Guillain-Barré, which causes weakness and generalized muscle paralysis [7]. ZIKV in pregnant women was associated with the outbreak of microcephaly in Brazilian newborns in 2016 [8].

Arboviruses can cause a variety of clinical presentations that range from mild to life threatening symptoms. However, the clinical condition of arbovirus infections is usually constituted by fever, headache, myalgia, arthralgia, and joint edema [9]. More than half of patients have skin rash associated with one of these classic symptoms [10]. Rash is any rash associated or not with mucosal lesions, fever and other symptoms, which may be a manifestation of infectious disease or an adverse reaction to a medication [11].

In arboviruses, the type of rash may vary according to the virus causing the disease, and it may appear between the first and twelfth day after the onset of the first symptoms [12,13]. In chikungunya infections, rashes occur in approximately 50% of patients, while in dengue and Zika, they may occur in between 50% to 82% and 90% to 100% of cases, respectively [14,15]. The implications of rashes on prognosis and clinical course of arboviruses are still unclear [10].

Other viral infections, such as HIV, parvovirus B19, Epstein-Barr mononucleosis and cytomegalovirus, rubella and measles, may also cause skin rashes, as well as bacterial infections, such as syphilis, whose classic palm-plantar rash appears in the secondary stage of the disease [9,16].

During 2018, there was a resurgence of measles cases in northern Brazil, which was mainly associated with the increase in Venezuelan immigrants combined with the low vaccination coverage, and contributed to the outbreak of cases in all age groups [17].

The current study aims to estimate the prevalence of arbovirus infection and other infectious causes in patients with acute skin rashes who were treated at a tertiary health unit in Manaus, Brazil.

## Methods

### Ethics statement

The study protocol was approved by the Ethics Review Board (ERB) of the Fundação de Medicina Tropical Doutor Heitor Vieira Dourado (FMT-HVD) under CAAE number 80961517.6.0000.0005 and protocol number 2.611.564, and followed the Guidelines and Norms Regulating Research on Human Subjects established in National Health Council Resolution 466/12/MS. Written informed consent was obtained from all the participants.

### Study area

Manaus (3° 1’ S, 60° 02’ W), located in the Brazilian Amazon (at the confluence of the Negro and Solimões Rivers), is the capital of the Amazonas state, in northern Brazil and has a territorial area of 11,401,092 km^2^. In 2018, its population was estimated at 2,145,444 inhabitants, the population density of the municipality was 158.06 inhab/km^2^ and the Municipal Human Development Index (HDI) was 0.737. It is the most populous city in the northern region and the seventh most populous in Brazil. The majority of the population (99.5%) lives in the urban areas of the municipality [18].

FMT-HVD, is a tertiary health unit for the diagnosis, treatment, and monitoring of tropical and infectious diseases in the Western Brazilian Amazon. Patients can seek medical assistance directly or be referred to the hospital from primary-level healthcare units.

### Study design and population

This is a cross-sectional study based on convenience sampling conducted with patients with acute rashes, with or without fever, who sought care at the FMT-HVD from February 2018 to May 2019. Individuals of either sex aged 18 years and over, who had skin rashes and the ability to provide formal consent were invited to voluntarily participate in the study. Pregnant women were excluded from the study.

### Sample collection and laboratory analyses

After voluntary consent, patients were seen by the physician and biological samples (blood and urine) were collected. Blood was obtained by venipuncture for HIV 1 and 2 immunochromatographic testing (MedTest HIV, MedLevensohn, Yuhang District, Hangzhou, China) and TR DPP-HIV 1e 2-SSP (Bio-Manguinhos, Rio de Janeiro, Brazil), and syphilis (Immuno-Fast Syphilis, REF 625020, Wama Diagnostic, São Carlos/SP, Brazil) testing. Positive results for HIV through immunochromatographic testing were confirmed by detection of anti-HIV antibodies using ELISA [19] and syphilis was confirmed by quantifying antibody titers against syphilis by VDRL, according to the protocol of the Brazilian Ministry of Health [20]. Hematological analyses were performed using automatic testing equipment (HORIBA ABX, PENTRA 120).

Blood plasma was used in serological and molecular biology techniques in order to evaluate infections caused by Zika, dengue, chikungunya, Mayaro, parvovirus B19, Epstein-Barr, cytomegalovirus, measles and rubella. Urine samples were used to evaluate Zika, chikungunya and measles infections. The hCG *QUICKSTRIP* rapid pregnancy test (Ebram, São Paulo, Brazil) was used to test women’s urine.

ZIKV, DENV, CHIKV, MAYV, OROV and measles infections were evaluated using RT-qPCR (real-time polymerase chain reaction) analysis of plasma and urine. A ZDC Kit (Bio-Manguinhos, Fiocruz), which is approved by the National Health Surveillance Agency/ANVISA (registration no. 80142170032), was used to evaluate ZIKV, DENV and CHIKV infection, while MAYV, OROV and measles infections were evaluated according to the methodologies described by Waggoner et al. [21], Naveca et al. [22] and Hummel et al. [23], respectively. The CT values were defined according to each of the previously mentioned diagnostic protocols.

Parvovirus B19, Epstein-Barr, cytomegalovirus, measles and rubella infections were assessed through qualitative detection of IgM class antibodies using ELISA and the Euroimmun kit (Euroimmun, Lübeck, Germany) according to the manufacturer’s instructions. Reagent and undetermined samples were revaluated using the electrochemiluminescence method. The commercial kit for the LIAISON analyzer (DiaSorin, Italy) was used to establish confirmation via immunodiagnosis.

### Definitions

Confirmed infection with dengue, chikungunya, Zika, Mayaro, or Oropouche was defined as a positive result by RT-qPCR for each one of these diseases.

Confirmed infection with parvovirus B19, rubella, mononucleosis caused by Epstein-Barr, and cytomegalovirus were defined as positive result after using the electrochemiluminescence method.

Confirmed infection of measles was defined as a positive IgM result in an immunodiagnostic test or a positive RT-qPCR result from a urine sample according to the criterion of the Brazilian Ministry of Health.

Confirmed infection with HIV and syphilis were defined as a positive result from immunochromatographic testing confirmed by detection of anti-HIV antibodies by ELISA and syphilis was confirmed by quantifying antibody titers against syphilis using VDRL, according to the protocol of the Brazilian Ministry of Health.

### Statistical analysis

All clinical and laboratory data obtained from patients were collected through standardized questionnaires and recorded using Epi Info software (version 7, CDC, Georgia, USA). Analysis was performed using Stata software version 13 (Stata Corp LP, Texas, USA). Descriptive analysis was presented by percentage distribution according to each category and mean and standard deviation were calculated for the continuous variables. The Pearson Chi-square test and Fisher exact test were used to determine the association between categorical variables. We used the *t* test to compare means in cases where the distributions were parametric; for non-parametric distribution, we used the Mann-Whitney test. The significance level defined was *p* < 0.05, and the confidence level was 95%.

## Results

**Fig 1:**
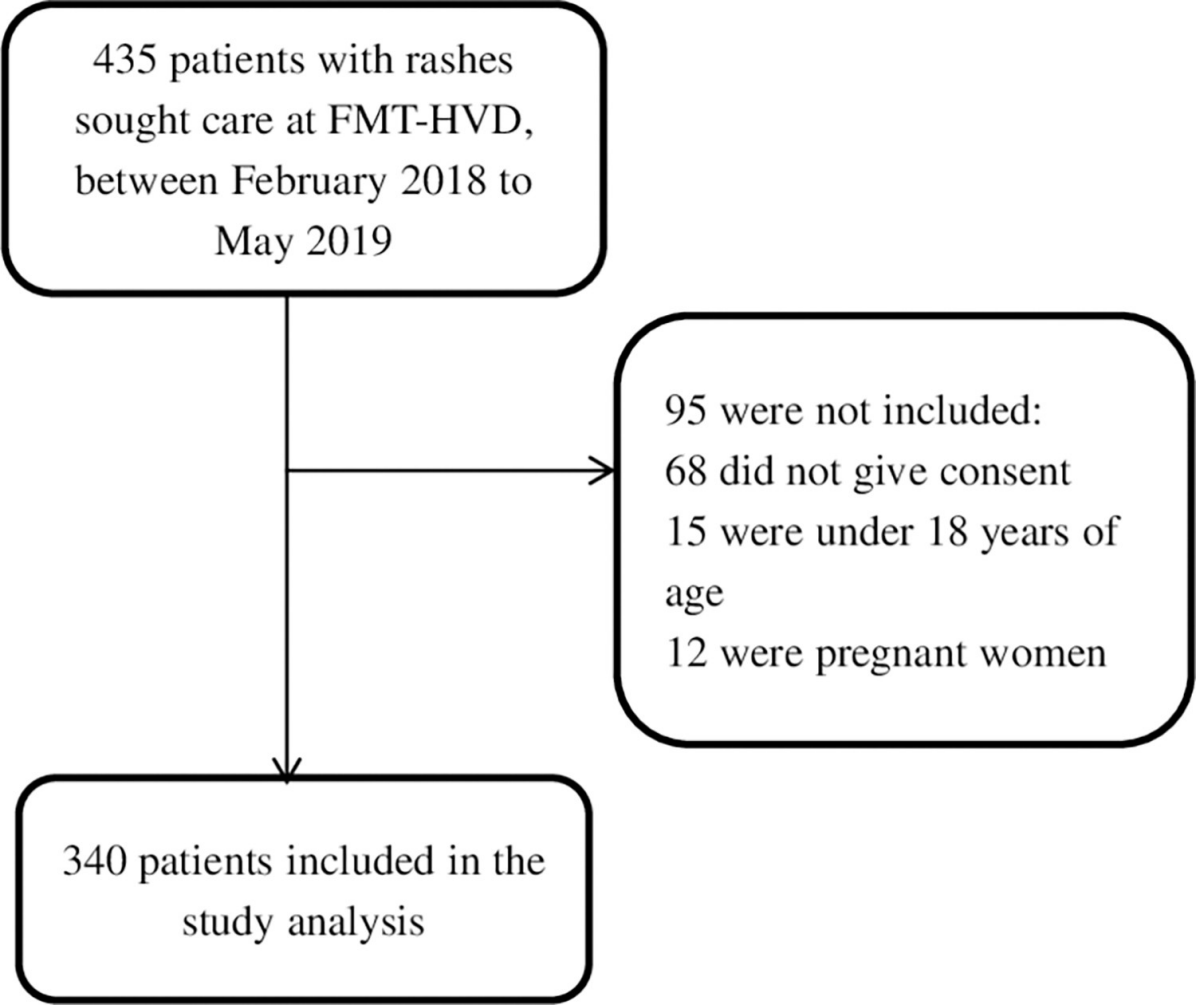
Flowchart of inclusion of patients treated at FMT-HVD for skin rash.

Between February 2018 and May 2019, a total of 435 patients with acute rash sought care at FMT-HVD, of which 340 were included in this study (Fig 1).

### Characteristics of the study population

See Table 1 for the baseline and clinical characteristics of all patients included in the study.

**Table 1:** Baseline and clinical characteristics of patients with rash, FMT-HVD, Manaus, February 2018 to May 2019.

Characteristics		Total (N = 340)
		n (%) or Mean ± SD^a^
**Age (years)**		36.5 ± 12.2
	18 to 40	226 (66.5)
	41 to 59	99 (29.1)
	≥ 60	15 (4.4)
**Gender**	Male	112 (39.9)
	Female	228 (67.1)
**Race**	White	40 (11.8)
	Black	3 (0.9)
	Brown	293 (86.2)
	Indigenous	1 (0.3)
	Other	3 (0.9)
**Days since onset of symptoms** ^**b**^		3.9 ± 2.4
	0 to 2	103 (30.3)
	3 to 5	163 (47.9)
	6 to 8	64 (18.8)
	≥ 9	10 (3.0)
**Signs and symptoms**	Maculopapular rash	305 (90.5)
	Pruritus	298 (87.6)
	Fever	172 (50.6)
	Edema	215 (63.2)
	Arthralgia	243 (71.5)
	Conjunctival hyperemia	191 (57.2)

^a^Mean ± standard deviation.

^b^Days from the onset of symptoms until medical attention and collection of laboratory specimens. Minimum value: 1 day of symptom; maximum value: 17 days of symptoms.

The average age of the patients was 36.5 years (SD ± 12.2 years, range 18 to 80 years), 67.1% were females (228/340) and 86.2% were brown-skinned (293/340). The most frequent signs and symptoms observed in all patients were a skin rash with a maculopapular characteristic (90.5%, 305/340), with a mean evolution of 2.3 days (SD ± 1.7 days) from the onset of symptoms until medical attention was sought at FMT-HVD. Pruritus (87.6%, 298/340), arthralgia (71.5%, 243/340), edema (63.2%, 215/340), conjunctival hyperemia (57.2%, 191/340) and fever (50.6%, 172/340) were the other sign and symptoms observed in the included patients. The mean time from the onset of general signs and symptoms to the moment of collection of biological material for diagnosis was 3.9 days (SD ± 2.4 days) in the population. By stratifying in a period of days, we observed that most patients (47.9%, 163/340) experienced 3 to 5 days of signs and symptoms, followed by the group of patients from 0 to 2 days of symptoms (30.3%, 103/340).

A similar pattern for baseline and clinical characteristics was observed with regard to the confirmed ZIKV cases (Table 2).

**Table 2:** Baseline and clinical characteristics of patients with Zika infection and other infectious causes of rash, FMT-HVD, Manaus, February 2018 to May 2019.

Characteristics		Zika positive (N = 222)	Other infections^a^ (N = 27)	
		n (%) or Mean ± SD^b^	n (%) or Mean ± SD	*p*-value^c^
**Age (years)** ^**d**^		38.4 ± 12.2	29.1 ± 7.3	0.001
**Age** ^**e**^	18 to 40	130 (58.6)	24 (88.9)	0.008
	41 to 59	81 (36.5)	3 (11.1)	
	≥ 60	11 (4.9)	0	
**Gender** ^**e**^	Male	68 (30.6)	12 (44.4)	0.189
	Female	154 (69.4)	15 (55.6)	
**Race** ^**e**^	White	29 (13.1)	1 (11.1)	1.000
	Black	3 (1.3)	0	
	Brown	186 (83.8)	24 (88.9)	
	Indigenous	1 (0.4)	0	
	Other	3 (1.4)	0	
**Days since onset of symptoms** ^**d**^ ^**f**^		3.8 ± 2.3	4.6 ± 2.3	0.0401
**Days since onset of symptoms** ^**e**^	0 to 2	66 (29.7)	4 (14.8)	0.140
	3 to 5	115 (51.8)	15 (55.6)	
	6 to 8	36 (16.2)	6 (22.2)	
	≥ 9	5 (2.3)	2 (7.4)	
**Signs and symptoms** ^**g**^	Maculopapular rash	203 (91.4)	25 (92.6)	0.839
	Pruritus	203 (91.4)	24 (88.9)	0.659
	Fever	117 (52.7)	17 (63.0)	0.313
	Edema	150 (67.6)	18 (66.7)	0.925
	Arthralgia	167 (75.2)	20 (74.1)	0.896
	Conjunctival hyperemia	146 (65.8)	13 (48.2)	0.072

^a^Other infections (DENV = 3, CHIKV = 1, parvovirus B19 = 3, measles = 10, HIV = 1, syphilis = 2, EBV + Parvovirus B19 = 1, CMV + Parvovirus B19 = 2, CMV + EBV + Parvovirus B19 = 3, total = 27).

^b^Mean ± standard deviation.

^c^Unadjusted *p*-value.

^d^Mann-Whitney test.

^e^Fisher exact test.

^f^Days from the onset of symptoms until medical attention and collection of laboratory specimens. Minimum value: 1 day in Zika and other infections positive patients. Maximum value: 16 days in Zika positive and 17 days in other infections positive patients.

^g^Pearson Chi-square test.

The average age of the ZIKV-positive patients was 38.4 years (SD ± 12.2 years, range 18 to 80 years), the majority were female (67.1%, 154/222) and brown-skinned (83.8%, 186/222). The mean quantity of days since the onset of symptoms the moment of collection of biological material for diagnosis in ZIKV-positive patients versus patients that tested positive for other infections was 3.8 ± 2.3 (minimum: 1 day maximum: 16 days) versus 4.6 ± 2.3 (minimum: 1 day, maximum: 17 days); *p* = 0.0401. We did not find significant statistical difference in the signs and symptoms between ZIKV-positive versus patients that tested positive for other infections.

See S1 Table for the baseline and clinical characteristics of all infection-negative patients included in the study. The characteristics of the monoinfections and co-infections-positive patients are described in the supporting information (S2 and S3 Tables), respectively.

### Frequency of arboviral infections

**Fig 2:**
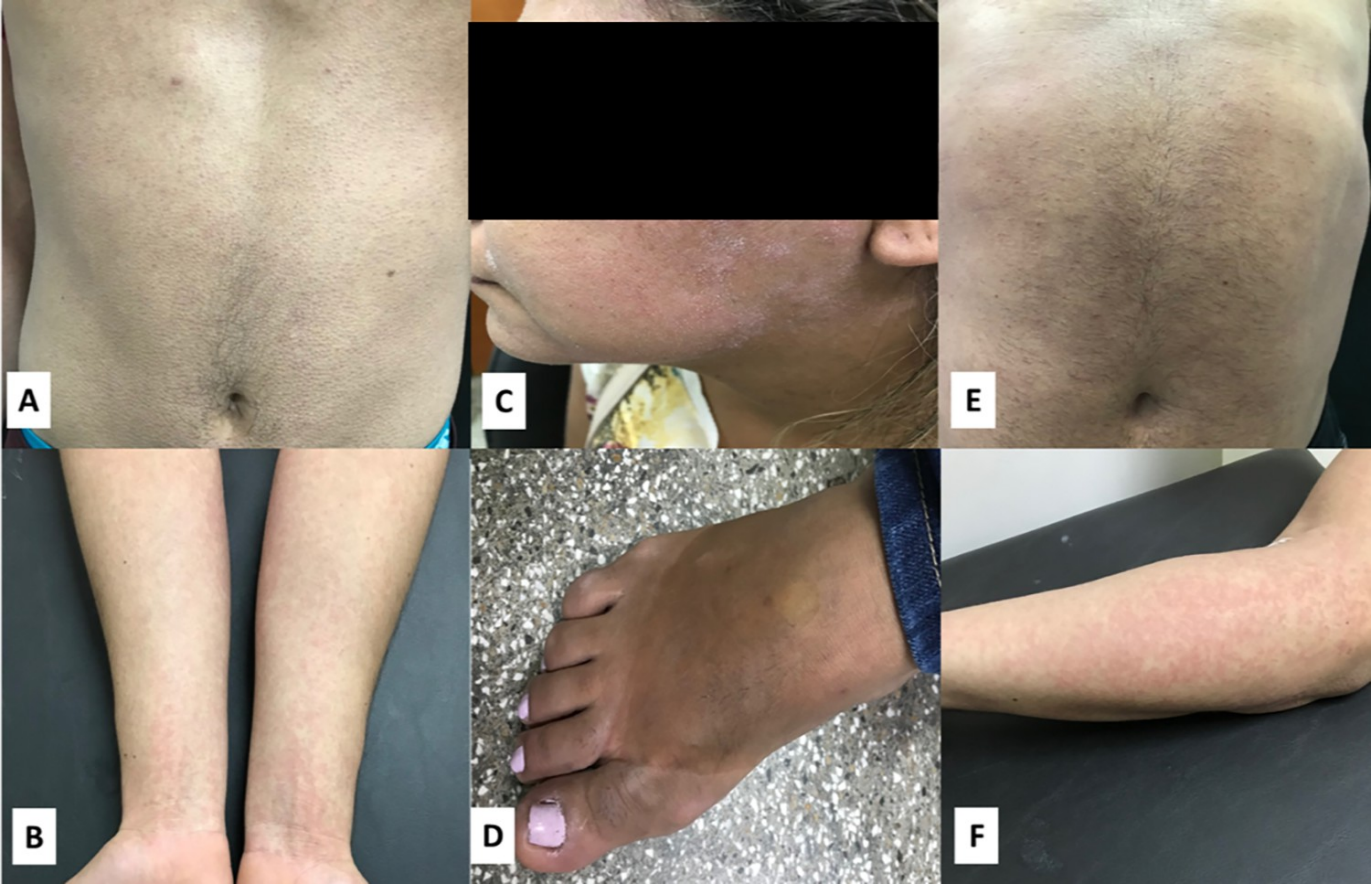
Rash observed in patients with diagnosis for arboviruses. (A and B) rash observed on thorax and upper limbs of ZIKV-positive patient. (C and D) peeling rash on face and lower limb edema of a CHIKV-positive patient. (E and F) rash observed on thorax, abdomen and upper limbs of a dengue-positive patient.

See Table 3 for the frequencies of arboviral infection. Out of the 340 samples that were tested, 67.9% (231/340) participants were confirmed as having one of the arboviral infections (ZIKV, DENV or CHIKV). The highest prevalence corresponded to ZIKV monoinfections (65.3%, 222/340). Three patients had confirmed dengue infection (0.9%, 3/340) and there was one case of CHIKV infection (0.3%, 1/340). No cases of MAYV and OROV infection were found. Most of the cases of arboviruses described in this work were diagnosed between February and May, and this occurred both in 2018 and 2019. See Fig 2 for the rashes observed in patients diagnosed with arboviruses.

**Table 3:** Prevalence of arboviruses and other infectious causes of rash and co-infections in patients treated at FMT-HVD, Manaus, between February 2018 to May 2019.

Monoinfections (N = 340)	n (%)
ZIKV	222 (65.3)
DENV	3 (0.9)
CHIKV	1 (0.3)
Parvovirus B19	3 (0.9)
Measles	10 (2.9)
HIV	1 (0.3)
Syphilis	2 (0.6)
**Co-infections**	
ZIKV + HIV	1 (0.3)
ZIKV + Measles	1 (0.3)
ZIKV + Parvovirus B19	1 (0.3)
ZIKV + EBV	1 (0.3)
EBV + Parvovirus B19	1 (0.3)
CMV + Parvovirus B19	2 (0.6)
CMV + Syphilis	1 (0.3)
ZIKV + EBV + Parvovirus B19	1 (0.3)
CMV + EBV + Parvovirus B19	3 (1.2)
Negative for all infections tested	86 (25.3)

EBV: Epstein-Barr; CHIKV: Chikungunya virus; CMV: Cytomegalovirus; DENV: Dengue virus; HIV: Human Immunodeficiency Virus; ZIKV: Zika virus.

### Frequency of other infectious diseases may also cause rash

**Fig 3:**
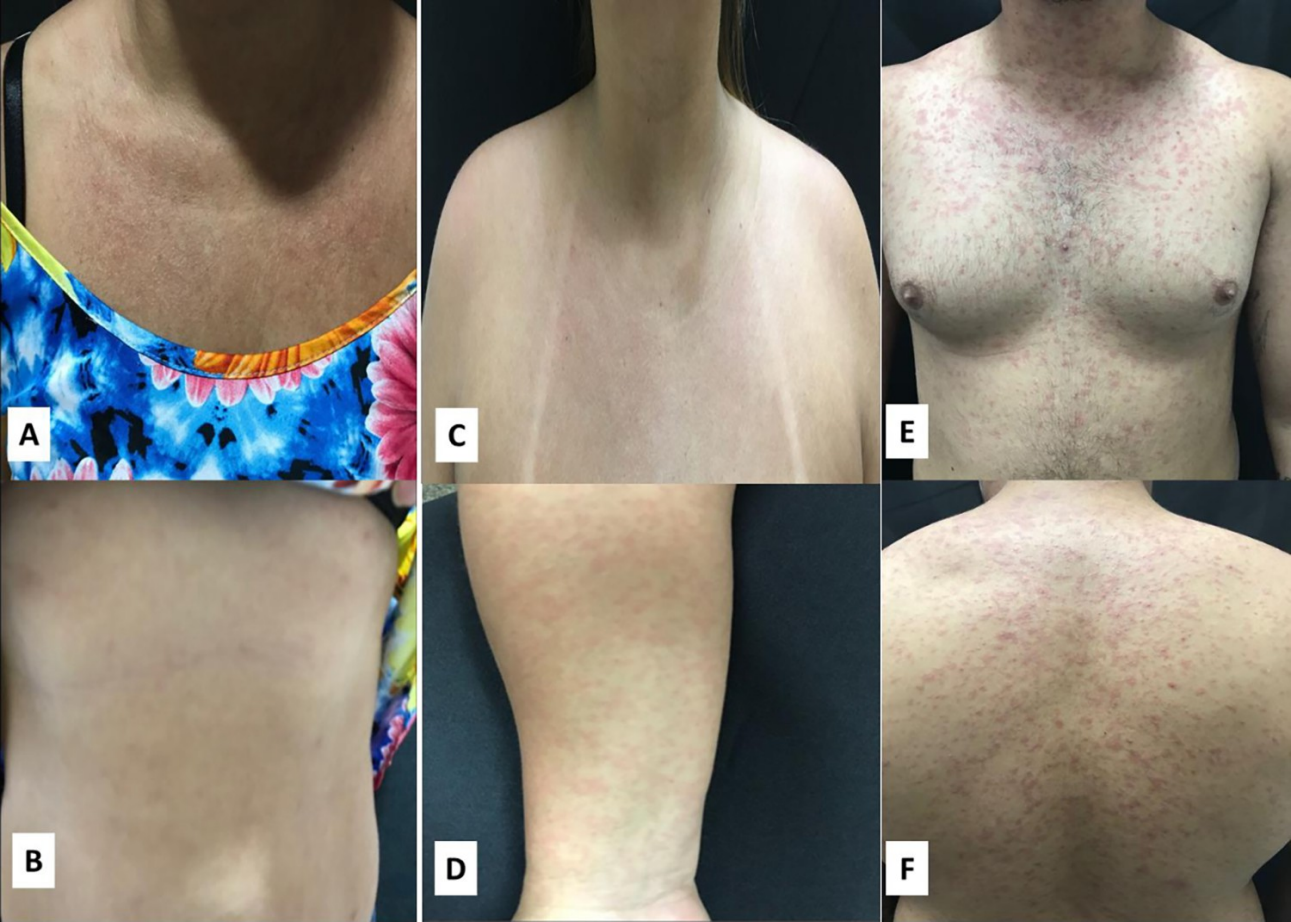
Rashes observed in patients diagnosed with other infectious diseases in this study. (A and B) rash observed on thorax and abdomen of an HIV-positive patient. (C and D) rash observed on thorax and upper limbs of a syphilis-positive patient. (E and F) rash observed on anterior and posterior thorax of a measles-positive patient.

See Table 3 for prevalence of arbovirus and other infectious diseases that may also cause rashes. Other monoinfectious diseases that were detected were parvovirus B19 (0.9%, 3/340), and measles (2.9%, 10/340). HIV monoinfections and syphilis accounted for 0.3% (1/340) and 0.6% (2/340), respectively. No cases of rubella, CMV and EBV mononucleosis were found. See Fig 3 for the rashes observed in patients diagnosed with other infectious diseases in this study.

### Frequency of co-infections

See Table 3 for frequency of co-infections. The co-infections identified were involved ZIKV+HIV (0.3%, 1/340); ZIKV+measles (0.3%, 1/340); ZIKV+parvovirus B19 (0.3%, 1/340); ZIKV+EBV (0.3%, 1/340). We also observed co-infections involving: EBV+parvovirus B19 (0.3%, 1/340); CMV+parvovirus B19 (0.6%, 2/340), CMV+syphilis (0.3%, 1/340); CMV+EBV+parvovirus (0.9%, 3/340) and ZIKV+EBV+parvovirus B19 (0.3%, 1/340).

A total of 25.3% (86/340) of patients were negative for all infectious agents tested in this study. The baseline and clinical characteristics of them are described in supporting information (S1 Table).

## Discussion

Our findings reveal that a total of 340 patients, 74.7% (256/340) tested positive for at least one of the diseases evaluated in this study. The highest frequencies corresponded to arbovirus monoinfections such as ZIKV (65.3%, 222/340), followed by other etiologies such as measles (2.9%, 10/340), DENV (0.9%, 3/340), parvovirus B19 (0.9%, 3/340) and CHIKV (0.3%, 1/340). HIV and syphilis monoinfections accounted for 0.3% (1/340) and 0.6% (2/340). Monoinfections by EBV and CMV were not observed.

Accordingly, in the context of screening for agents that cause rash, the frequency of ZIKV was higher. Arboviruses have a variety of signs and symptoms that are often confused with other infectious diseases. Therefore, many cases can be clinically misdiagnosed. All participants included in this study had a clinical history that was suggestive of arbovirus infection. However, we also investigated and found other causes of skin rash due to the possibility that other infectious agents were responsible for these febrile and non-febrile illnesses.

The participants of this study were mostly women with an average age of 36 years, which demonstrates that this would potentially be the gender and age group most affected by arboviruses. Other studies have also found similar results and have suggested that women are possibly more affected by arboviruses because they are in the home environment for longer periods and, since transmission is mainly at home and in peridomestic areas, the difference may be justified by the higher exposure, or because they tend to be more inclined to seek the health services [24,25]. The high prevalence of ZIKV among women of childbearing highlights the importance of prevention measures in this group in order to avoid adverse outcomes such as congenital Zika syndrome.

Regarding the prevalence of arboviruses, ZIKV monoinfection presented a high prevalence (65.3%, 222/340). High ZIKV prevalence has already been observed in several states of Brazil, as this disease was in a state of outbreak, especially in the southeast and northeast [26]. Few studies confirmed cases of ZIKV in northern Brazil, though one recent study reported a frequency of 4.3% (7/164) of confirmed cases of ZIKV in the state of Rondônia [27]. Factors such as large population displacements, the presence of vectors and hot and humid climate conditions are conducive to the spread of diseases such as ZIKV in the Amazon region [28], and these aspects directly affect the seasonality of arboviruses, which generally occur mainly during the rainy periods of the year [29], and this was an aspect that was also evidenced throughout this study. In Brazilian cities like Manaus, where DENV is already highly endemic, the introduction of ZIKV puts thousands of people at risk of co-infection with these two or more potentially highly pathogenic arboviruses, since CHIKV, MAYV and OROV are also responsible for cases in this region [30].

DENV and CHIKV presented low prevalence in this study. Chaves et al. [30] used experimental *Aedes aegypti* co-infections with DENV and ZIKV strains to demonstrate that this mosquito population has a higher efficiency and a preference for transmitting ZIKV in DENV co-infections in the vector, which may justify the low prevalence of DENV and CHIKV during the period of the ZIKV outbreak in Manaus.

DENV outbreaks have already been described in the last twenty years in the city of Manaus, and the last one occurred in 2011. That year, a prevalence of 38.4% (260/677) of cases was detected in the same tertiary health unit where we conducted our study. Furthermore, thirteen patients were co-infected with more than one DENV serotype and six (46.1%) of them had a more severe clinical presentation of the disease. Numerous serious cases were recorded, as well as the proof of circulation of the four serotypes of the disease in the region [31,32].

In our study, no cases of MAYV and OROV infection were found. The last outbreaks of MAYV and OROV in Manaus were recorded in 2007 and 2008, which included three records of severe manifestations of central nervous system infections by OROV [33–35]. In 2018, in the city of Tefé, in the state of Amazonas, Brazil, nine cases (30%, 9/30) of human OROV infection were confirmed, thus proving the continuity of virus circulation and autochthonous transmission in the state of Amazonas [36]. It is important to investigate these viruses since fever (with/without skin rash) caused by OROV, for example, is less well studied, but it is believed to have caused more than 500,000 febrile infections in Brazil in recent decades. Such cases could easily be clinically misdiagnosed as dengue infections [37].

Our study revealed some cases of co-infections. It has already been mentioned that we used only IgM antibody quantification to diagnose some of the rash-causing diseases (CMV, EBV, parvovirus B19 and measles). Most of the co-infections in this study involved these viruses. The simultaneous appearance of IgM that was reactive for more than one virus is not a new phenomenon in diagnostic virology. In these cases, for example, studies indicate that a primary infection can be confirmed by antibody avidity as another immunodiagnosis option. Avidity tests will confirm a primary infection by detecting low avidity specific IgG or rule out a primary infection by detecting high avidity specific IgG [38]. However, we did not perform an avidity test since this methodology is not performed in the hospital routine where we developed our study. Despite this, any indeterminate or positive IgM samples were reevaluated using the electrochemiluminescence method for diagnostic confirmation.

In the study population, a single ZIKV and HIV co-infection was evidenced. Information on the influence of ZIKV infection on the clinical course of HIV is scarce and requires studies that are able to clarify whether HIV infection can worsen the clinical course of ZIKV infection or vice versa [39].

We also observed a single ZIKV and measles co-infection, and this finding is important because, during arbovirus outbreaks, paying attention only to morbilliform rash can mask the possible diagnosis of other infectious agents such as measles. The availability of serological tests, a complete medical history, and physical examinations are an important part in the diagnosis and the screening of patients for common infections such as measles and should be considered during an arbovirus outbreak as this infection still remains endemic in several parts of the world, as well as in places where diseases were reintroduced, as in the case of Brazil. Other co-infections including ZIKV were ZIKV+parvovirus B19 (0.3%, 1/340) and ZIKV+EBV (0.3%, 1/340). The interactions between these diseases should be studied in greater depth.

We also noticed measles cases (2.9%, 10/340), which is an infectious disease that has supposedly been eliminated from Brazil [40]. During 2018, there was a resurgence of cases in Brazil, mainly associated with the increase in Venezuelan immigrants, and which marked the reintroduction of the disease to Brazil, with cases initially confirmed in the state of Roraima [41]. In 2018, in the state of Amazonas, more than 8,000 suspected cases were reported and about 1,000 cases were initially confirmed [17].

Classically, measles is characterized by an acute febrile illness associated with a erythematous, maculopapular rash. The illness begins with fever and typically at least one of the three “Cs”: cough, coryza, and conjunctivitis. Koplik’s spots appear on the buccal mucosa as small white papules and provide an opportunity to clinically diagnose measles a day or two before the rash. The rash appears 3–4 days after the onset of fever, first on the face and behind the ears, and then spreads to the trunk and extremities, coinciding with development of the adaptive immune response. The fever and catarrhal symptoms typically peak with the rash, which persists for 3–4 days [42].

In our study, patients diagnosed with measles had mild symptoms, many of which resembled symptoms of arboviruses, and, at the time of care, they did not have the classic flu-like symptoms (cough and coryza). The average of the days since the onset of symptoms to the time of medical care and collection of specimens for laboratory diagnosis was 4.3 days (SD ± 2. 5 days). Most measles-positive patients had between 3 to 5 days (60.0%, 6/10). Many of the cases were treated in the first months of 2018, which was the beginning of the measles outbreak in Manaus, and a time when there was no efficient screening system for suspected cases in the city. As such, some mild cases, such as those in this study, may have received other diagnoses of exanthematic diseases that were not measles. However, during the outbreak period, this study showed a significant prevalence of the disease, which can be explained by the establishment of a screening and care system for suspected measles cases in the unit where we carried out our study. This was right after the disease outbreak since it is the reference institution for infectious diseases in Manaus.

Sexually transmitted infections, such as HIV and syphilis, are also responsible for rashes [11,13], and these are aspects that we also evidenced in the study population. In the case of HIV, rash is usually one of the manifestations of the acute phase of infection, usually associated with fever, weight loss and flu-like symptoms [43]. Rashes due to syphilis, however, are described as one of the possible manifestations of the secondary phase of the disease, and may occur in up to 80% of cases [16]. Syphilis is an infectious disease that can manifest itself in many ways, owing to its varied and often subtle manifestations that can mimic other infections [44].

The rash of secondary syphilis can be widespread or localized; pustular, macular, papular, or scaly in appearance; and might mimic other dermatological processes including pityriasis rosea, psoriasis, drug eruptions. Symptoms like malaise, myalgia, sore throat, headache or low-grade fever can commonly occur, but are not so frequent. Due to its multiple forms of clinical manifestations, many clinicians may not initially suspect the disease. Without treatment, lesions of secondary syphilis can disappear spontaneously without scars. Resolution of untreated manifestations of secondary syphilis can take weeks to several months [45]. In our study, the two syphilis monoinfection (0.6%, 2/340) patients diagnosed with the disease had an average of 2 days of symptoms (SD ± 1) and presented a clinical picture that was not similar to the classic secondary syphilis picture that is described in the literature. We had one co-infection of syphilis and CMV (0.3%, 1/340). Few cases of this disease were detected in this study due to the screening system for sexually transmitted syphilis infections in the health unit where we conducted the study. Thus, patients with classic illnesses were soon diagnosed and treated, and did not undergo outpatient triages for diseases such as arboviruses.

Among the other causes of rash in the evaluated patients, parvovirus B19 monoinfection (0.9%, 3/340) was observed. Most cases of parvovirus B19, for example, are described in the child population, but some studies demonstrate the occurrence of cases in adult individuals. In a study carried in Campinas, Brazil, that investigated etiologies of illnesses that cause rashes and fever showed a frequency of 2.5% (30/519) of parvovirus B19 infection. In another study conducted in Cuba, the frequency of rashes caused by parvovirus B19 was 10.7% (32/298), and 11 positive cases (34.4%) were in people from 20 years and older (*p* < 0.05) [46]. The identification of parvovirus B19 as a causative agent of rash and fever in Manaus was first described in 1999, in which 27 cases (2.43%) were reported during that year’s DENV outbreak [47].

As previously mentioned, all patients had skin rash and clinical history of arbovirus infection. However, 25.3% (86/340) of patients were negative for all infectious agents tested in this study. We believed that most of these participants who tested negative for all infections would be infected with an arbovirus, as they presented clinical signs that were suggestive of these diseases at the time of inclusion, but the method employed was not able to detect a possible low viral load in plasma and urine. Some studies reveal that, in diseases such as ZIKV, high viral load titers can be detected in other body fluids for a period greater than or equal to those found in urine. Shedding of ZIKV RNA in saliva and urine from a female patient until 29 days after the onset of symptoms was reported in Italy in 2016 [48]. Another study, which was performed in Florida, revealed a higher rate of ZIKV RNA detection in saliva and urine (ranging from 81% to 92%, respectively) when compared to the RNA level detected in the serum (51%) [49].

Shedding in saliva and urine has also been demonstrated for other vector-borne flaviviruses, such as dengue [50,51] and West Nile virus [52]. In addition, CHIKV, has been isolated in oral fluids of patients with severe infection and in the saliva of experimentally infected mice and monkeys [53]. Our protocol used for the diagnosis of ZIKV and CHIKV was developed to detect these viruses in plasma and urine. However, due to the scientific evidence of long viral persistence of these viruses in other body fluids such as saliva, it is suggested that this fluid may also be used as an alternative in the routine diagnosis of arboviruses. Furthermore, we do not know whether there are other arboviruses, such as Saint Louis or West Nile, for example, that are circulating in the Brazilian Amazon, so studies that investigate these arboviruses should be carried out.

We cannot rule out the possibility that these 25.3% undiagnosed patients presented an initial picture of other diseases that were not infectious. There is a possibility that skin rash associated with fever or without fever is indicative of a possible rheumatological disease, for example. Furthermore, the possibility of other circulating viral diseases that cause acute rash (though not included in this series) cannot be excluded.

This study has some limitations in such as it may not be feasible to recommend routine testing for all fever-rash cases as broadly as was done in this study because of the high costs and difficulties associated with this method. There is also the possibility of cross-reactions in the serological tests used, though the use of avidity tests can confirm a primary infection as a diagnostic option, or molecular biology techniques followed by genetic sequencing would bring greater reliability to the results. Furthermore, the infectious diseases detected in this work are due to the FMT-HVD being a health care facility that serves the population of Manaus through spontaneous demand, as well as through referencing done by other health facilities in the city Manaus and the interior of the state of Amazonas. Some people with cases of rashes undoubtedly remained at home or received clinical diagnosis in other health units in Manaus. It is important to highlight the role of FMT-HVD as a sentinel health unit in the monitoring and laboratory diagnosis of numerous health problems in the western Amazon region of Brazil.

The detection of different rash-causing pathogens provides valuable feedback to clinicians and patients. The positive cases of diseases such as measles show that vaccination policies should be strengthened for immune preventable diseases such as measles, as well as the implementation of better vector control strategies for decreasing the number of cases of arboviruses. Furthermore, it is worth mentioning that sexually transmitted infections, such as HIV and syphilis, are important causes of rashes and should be remembered at the time of clinical care.

## Conclusion

The results reveal that 74.7% (254/340) of the patients enrolled in the study were positive for one or more of the infections evaluated by us. The highest prevalence was for ZIKV monoinfection in rash patients who participated. Although low prevalence of DENV and CHIKV has been observed, these cases cannot be underestimated. No cases of MAYV, OROV or rubella were found. Our findings may reflect regional variations and important causes of rash, like measles, HIV and syphilis. However, they may also be influenced by limitations in testing, as well as a low viral load at the time of sample collection. For an endemic region for arborviruses, among other viruses that cause acute rash, a good alternative for surveillance actions would be the use of serological tests for screening in periods of greater rainfall; a period in which there are higher frequencies of cases such as DENV and ZIKV, for example, in addition to ongoing monitoring changes in diseases dynamics over the time. The results herein may aid surveillance and control strategies for diseases such as arboviruses and other diseases that cause manifestations of skin rash.

## Supporting information

S1 Table Baseline and clinical characteristics of patients with Zika infection and patients who tested negative for all infectious diseases, FMT-HVD, Manaus, February 2018 to May 2019. ^a^Mean ± Standard deviation. ^b^Unadjusted *p*-value. ^c^Mann-Whitney test. ^d^Fisher exact test. ^e^Minimum value: 1 day of symptom in the general population group, Zika positive group and negative group for all rash-causing infectious diseases tested in this study. Maximum value: 17 days of symptoms in the general population group, 16 days in the Zika positive group and 17 days in the negative group for all diseases tested.
(DOCX)

S2 Table Baseline and clinical characteristics of patients with laboratory-confirmed monoinfections by arboviruses and other infectious causes of rash, FMT-HVD, Manaus, February 2018 to May 2019. ^a^Mean ± standard deviation.
(DOCX)

S3 Table Baseline, clinical and hematological characteristics of patients with laboratory-confirmed co-infections by arboviruses and other infectious causes of rash, FMT-HVD, Manaus, February 2018 to May 2019. EBV: Epstein-Barr; CHIKV: Chikungunya virus; CMV: Cytomegalovirus; HIV: Human Immunodeficiency Virus; ZIKV: Zika virus. ^a^Mean ± standard deviation.
(DOCX)

S1 File STROBE Statement—Checklist of items that should be included in reports of cross-sectional studies. (PDF)

S2 File Dataset. (XLSX)

## References

[1] Weaver SC, Reisen WK. Present and Future Arboviral Threaths. Antiviral Research. 2010;85(2):1–36. 19857523 10.1016/j.antiviral.2009.10.008 PMC2815176

[2] Higuera A, Ramírez JD. Molecular epidemiology of dengue, yellow fever, Zika and Chikungunya arboviruses: An update. Acta Trop [Internet]. 2019;190:99–111. doi: 10.1016/j.actatropica.2018.11.010 30444971

[3] Lima-Camara TN. Emerging arboviruses and public health challenges in Brazil. Rev Saude Publica. 2016;50:1–7. 27355468 10.1590/S1518-8787.2016050006791 PMC4936892

[4] Liliana F, Vega R, Maria J, Bezerra T, Fabiano R, Said DC, et al. Emergence of chikungunya and Zika in a municipality endemic to dengue, Santa Luzia, MG, Brazil, 2015–2017. Rev. Soc. Bras. Med. Trop. 2019;1–9. 10.1590/0037-8682-0347-2018 30652797

[5] Laserna A, Barahona-correa J, Baquero L, Castañeda-cardona C, Rosselli D. Economic impact of dengue fever in Latin America and the Caribbean: a systematic review. Rev Panam Salud Publica. 2018;42:1–11. doi: 10.26633/RPSP.2018.111 31093139 PMC6386068

[6] Teich V, Fahham. Aedes aegypti e sociedade: o impacto econômico das arboviroses no Brasil. J Bras Econ Saúde. 2018;9(3):267–76.

[7] Oehler E, Watrin L, Larre P, Leparc-Goffart I, Lastre S, Valour F, et al. Zika virus infection complicated by guillain-barr syndrome—case report, French Polynesia, December 2013. Eurosurveillance. 2014;19(9):7–9. 10.2807/1560-7917.es2014.19.9.20720 24626205

[8] Oliveira Melo AS, Malinger G, Ximenes R, Szejnfeld PO, Alves Sampaio S, Bispo De Filippis AM. Zika virus intrauterine infection causes fetal brain abnormality and microcephaly: Tip of the iceberg? Ultrasound Obstet Gynecol. 2016;47(1):6–7. doi: 10.1002/uog.15831 26731034

[9] Korman AM, Alikhan A, Kaffenberger BH. Viral exanthems: An update on laboratory testing of the adult patient. J Am Acad Dermatology [Internet]. 2017;76(3):538–50. doi: 10.1016/j.jaad.2016.08.034 28413059

[10] Huang H, Tseng H, Lee C, Chuang H, Lin S. Clinical significance of skin rash in dengue fever: A focus on discomfort, complications, and disease outcome. Asian Pacific Journal of Tropical Medicine. 2016;1–6. 10.1016/j.apjtm.2016.05.013 27393104

[11] Drago F, Ciccarese G, Gasparini G, Cogorno L, Javor S, Toniolo A, et al. Contemporary infectious exanthems: an update. Future Microbiol. 2017;12:171–193 doi: 10.2217/fmb-2016-0147 27838923

[12] Lupi O, Tyring SK. Tropical dermatology: Viral tropical diseases. J Am Acad Dermatol. 2003 Dec;49(6):979–1000. doi: 10.1016/s0190-9622(03)02727-0 14639375 PMC9628903

[13] Coelho S, Cestari T, Allen SH, Ramos M. Viral exanthems in the tropics. Clin Dermatol. 2007;25(2):212–20. doi: 10.1016/j.clindermatol.2006.05.009 17350501

[14] Inamadar AC, Palit A, Sampagavi V V, Raghunath S, Deshmukh NS. Tropical medicine rounds Cutaneous manifestations of chikungunya fever: observations made during a recent outbreak in south India. 2008. Int J Dermatol. 2008;47(2):154–9. 18211486 10.1111/j.1365-4632.2008.03478.x

[15] Hamel R, Liégeois F, Wichit S, Pompon J, Diop F, Talignani L, et al. Zika virus: epidemiology, clinical features and host-virus interactions. Microbes Infect [Internet]. 2016;18(7–8):441–9. doi: 10.1016/j.micinf.2016.03.009 27012221

[16] Tan C, Zhu WY. Arthralgia and scaly rashes over the palms and the soles. Brazilian J Infect Dis [Internet]. 2016;20(5):505–6. doi: 10.1016/j.bjid.2016.07.007 27542870 PMC9425497

[17] Elidio GA, de França GVA, Pacheco FC, Ferreira MM, Pinheiro JDS, Campos EN, et al. Measles outbreak: Preliminary report on a case series of the first 8,070 suspected cases, Manaus, Amazonas state, Brazil, february to november 2018. Eurosurveillance. 2019;24(2):1–8. doi: 10.2807/1560-7917.ES.2019.24.2.1800663 30646975 PMC6337055

[18] Instituto Brasileiro de Geografia e Estatística (IBGE). Cidades [Internet]. [Internet]. 2019 [cited 2019 Dec 2]. Available from: https://cidades.ibge.gov.br/brasil/am/manaus/panorama.

[19] Brasil. Ministério da Saúde. Secretaria de Vigilância em Saúde. Departamento de Vigilância, Prevenção e Controle das Doenças Sexualmente Transmissíveis, Aids e Hepatites Virais. Manual técnico para o diagnóstico da infecção pelo HIV / Ministério da Saúde, Secretaria de Vigilância em Saúde, Departamento de Vigilância, Prevenção e Controle das Doenças Sexualmente Transmissíveis, Aids e Hepatites Virais.– 2. ed.–Brasília: Ministério da Saúde, Brasil, 2015.

[20] Brasil. Ministério da Saúde. Secretaria de Vigilância em Saúde. Departamento de Vigilância, Prevenção e Controle das Doenças Sexualmente Transmissíveis, Aids e Hepatites Virais. Manual Técnico para Diagnóstico da Sífilis / Ministério da Saúde, Secretaria de Vigilância em Saúde, Departamento de Vigilância, Prevenção e Controle das Doenças Sexualmente Transmissíveis, Aids e Hepatites Virais.–Brasília: Ministério da Saúde, Brasil, 2016.

[21] Waggoner JJ, Rojas A, Mohamed-Hadley A, de Guillén YA, Pinsky BA. Real-time RT-PCR for Mayaro virus detection in plasma and urine. J Clin Virol. 2018;98:1–4. doi: 10.1016/j.jcv.2017.11.006 29172075 PMC5742299

[22] Naveca FG, Nascimento VAD, Souza VC, Nunes BTD, Rodrigues DSG, Vasconcelos PFDC. Multiplexed reverse transcription real-time polymerase chain reaction for simultaneous detection of Mayaro, Oropouche, and Oropouche-like viruses. Mem Inst Oswaldo Cruz. 2017 Jul;112(7):510–513. doi: 10.1590/0074-02760160062 28591313 PMC5452489

[23] Hummel KB, Lowe L, Bellini WJ, Rota PA. Development of quantitative gene-specific real-time RT-PCR assays for the detection of measles virus in clinical specimens. J Virol Methods. 2006;132(1–2):166–73. doi: 10.1016/j.jviromet.2005.10.006 16274752

[24] Colombo TE, Estofolete CF, Reis AFN, da Silva NS, Aguiar ML, Cabrera EMS, et al. Clinical, laboratory and virological data from suspected ZIKV patients in an endemic arbovirus area. J Clin Virol. 2017;96:20–5. doi: 10.1016/j.jcv.2017.09.002 28918127

[25] Cavalcante WD, Grande C, Grande C, Grande C, Grande C. Características epidemiológicas da dengue na comunidade São Januário II na cidade de Campina Grande–PB Epidemiological characteristics of dengue in the community Januário II in the city of Campina. Brazilian Journal of Pharmacy. 2011;92(4):287–94.

[26] Brasil P, Calvet GA, Siqueira AM, Wakimoto M, de Sequeira PC, Nobre A, Quintana Mde S, Mendonça MC, Lupi O, de Souza RV, Romero C, Zogbi H, Bressan Cda S, Alves SS, Lourenço-de-Oliveira R, Nogueira RM, Carvalho MS, de Filippis AM, Jaenisch T. Zika Virus Outbreak in Rio de Janeiro, Brazil: Clinical Characterization, Epidemiological and Virological Aspects. PLoS Negl Trop Dis. 2016;10(4). 10.1371/journal.pntd.0004636 PMC4829157 27070912

[27] Vieira DS, et al. Epidemiological profile of Zika, Dengue and Chikungunya virus infections identified by medical and molecular evaluations in Rondonia, Brazil. Rev. Inst. Med. trop. 2019; 61(40):1–6. doi: 10.1590/S1678-9946201961040 31432989 PMC6710006

[28] Niemeyer B, Ribeiro DF, Muniz BC, Gasparetto EL, Ventura N, Marchiori E. Síndrome congênita pelo vírus Zika e achados de neuroimagem: o que sabemos até o momento ?. Radio Bras. 2017;50(2):314–22. 10.1590/0100-3984.2017.0098 PMC5656072 29085165

[29] Agarwal A, Parida M, Dash PK. Impact of transmission cycles and vector competence on global expansion and emergence of arboviruses. Rev Med Virol. 2017;27(5):1–12. doi: 10.1002/rmv.1941 28857363

[30] Chaves BA, Orfano AS, Nogueira PM, Rodrigues NB, Campolina TB, Nacif-Pimenta R, et al. Coinfection with zika virus (ZIKV) and dengue virus results in preferential ZIKV transmission by vector bite to vertebrate host. J Infect Dis. 2018;218(4):563–71. doi: 10.1093/infdis/jiy196 29659904 PMC6047447

[31] Alves C, Bastos MDS, Ramasawmy R, Gimaque L, Silva W, Braga M, et al. Clinical and Virological Descriptive Study in the 2011 Outbreak of Dengue in the Amazonas, Brazil. Plos ONE. 2014;9(6). 10.1371/journal.pone.0100535 PMC4076277 24978469

[32] Maria R, Figueiredo P De, Naveca FG, Bastos MDS, Viana SDS, Gomes MP, et al. Dengue Virus. Emerging Infectious Diseases. 2008;14(4):667–9. 18394292 10.3201/eid1404.071185 PMC2570911

[33] Paula M, Moura G, Bastos MDS, Figueiredo RP De, Mara C, Oliveira C De. Mayaro fever in the city of Manaus, Brazil, 2007–2008. Vector borne and zoonotic diseases. 2012;12(1):2007–8. 10.1089/vbz.2011.0669 PMC3249893 21923266

[34] Mourão MP, Bastos MDS, Gimaque JB, Mota BR, Souza GS. Grimmer GS, Galusso ES, Arruda E, Figueiredo LT. Oropouche Fever Outbreak, Manaus. Emerging Infectious Diseases. 2009;15(12):2063–4. 19961705 10.3201/eid1512.090917 PMC3044544

[35] Bastos MDS, Figueiredo LTM, Naveca FG, Monte RL, Lessa N, De Figueiredo RMP, et al. Short report: Identification of oropouche Orthobunyavirus in the cerebrospinal fluid of three patients in the Amazonas, Brazil. Am J Trop Med Hyg. 2012;86(4):732–5. 22492162 10.4269/ajtmh.2012.11-0485 PMC3403753

[36] Naveca FG, Nascimento VA, Souza VC, Figueiredo RMd. Human Orthobunyavirus Infections, Tefé, Amazonas, Brazil. PLOS Currents Outbreaks. 2018;1:1–7. doi: 10.1371/currents.outbreaks.7d65e5eb6ef75664da68905c5582f7f7 29623245 PMC5878103

[37] Travassos Da Rosa JF, De Souza WM, De Paula Pinheiro F, Figueiredo ML, Cardoso JF, Acrani GO, et al. Oropouche virus: Clinical, epidemiological, and molecular aspects of a neglected orthobunyavirus. Am J Trop Med Hyg. 2017;96(5):1019–30. doi: 10.4269/ajtmh.16-0672 28167595 PMC5417190

[38] Thomas HIJ, Barrett E, Hesketh LM, Wynne A, Morgan-Capner P. Simultaneous IgM reactivity by EIA against more than one virus in measles, parvovirus B19 and rubella infection. J Clin Virol. 1999;14(2):107–18. doi: 10.1016/s1386-6532(99)00051-7 10588453

[39] Rothan HA, Bidokhti MRM, Byrareddy SN. Current concerns and perspectives on Zika virus co-infection with arboviruses and HIV. J Autoimmun. 2018;89:11–20. doi: 10.1016/j.jaut.2018.01.002 29352633 PMC5902419

[40] Lemos DRQ, Franco AR, de Sá Roriz MLF, Carneiro AKB, de Oliveira Garcia MH, de Souza FL, et al. Measles epidemic in Brazil in the post-elimination period: Coordinated response and containment strategies. Vaccine [Internet]. 2017;35(13):1721–8. doi: 10.1016/j.vaccine.2017.02.023 28256359

[41] Meneses CAR, Do Nascimento VA, De Souza VC, Maito RM, Gomes MA, Brígida Cunha CRS, et al. Molecular characterisation of the emerging measles virus from Roraima state, Brazil, 2018. Mem Inst Oswaldo Cruz. 2019;114(2):6–9. 10.1590/0074-02760180545 PMC6423661 30892375

[42] Moss WJ. Measles. Lancet. 2017;390(10111):2490–502. doi: 10.1016/S0140-6736(17)31463-0 28673424

[43] Khambaty MM, Hsu SS. Dermatology of the Patient with HIV. Emerg Med Clin North Am [Internet]. 2010;28(2):355–68. doi: 10.1016/j.emc.2010.01.001 20413018

[44] Peeling RW, Mabey D, Kamb ML, Chen X, David J, Benzaken AS, et al. HHS Public Access: Syphilis. Nat Rev Dis Prim. 2018 Oct;3(17073):49. 10.1038/nrdp.2017.73 PMC5809176 29022569

[45] Hook EW. Seminar Syphilis. Lancet. 2017;389:1550–7. 27993382 10.1016/S0140-6736(16)32411-4

[46] Ribas MDLÁ, Muller CP, Hübschen JM. Identification of human parvovirus B19 among measles and rubella suspected patients from Cuba. J Med Virol. 2019;91(7):1351–1354. doi: 10.1002/jmv.25444 30817853

[47] Figueiredo RMP, Thatcher BD, Lima ML De, Almeida TC, et al. Doenças exantemáticas e primeira epidemia de dengue ocorrida em Manaus, Amazonas, no período de 1998–1999. Exanthematous diseases and the first epidemic of dengue to occur. Rev. Soc. Bras. Med. Trop. 2004; 37(6). 10.1590/s0037-86822004000600009 15765597

[48] Barzon L, Pacenti M, Berto A, Sinigaglia A, Franchin E, Lavezzo E, et al. Isolation of infectious Zika virus from saliva and prolonged viral RNA shedding in a traveller returning from the Dominican Republic to Italy, January 2016. Euro Surveill. 2016;21(10):30159. doi: 10.2807/1560-7917.ES.2016.21.10.30159 26987769

[49] Bingham AM, Cone M, Mock V, Heberlein-larson L, Stanek D. Comparison of Test Results for Zika Virus RNA in Urine, Serum, and Saliva Specimens from Persons with Travel-Associated Zika Virus Disease—Florida, 2016. MMWR Morb Mortal Wkly Rep. 2016; 65(18):475–8. doi: 10.15585/mmwr.mm6518e2 27171533

[50] Andries A, Duong V, Ly S, Cappelle J, Kim KS, Try PL, et al. Value of Routine Dengue Diagnostic Tests in Urine and Saliva Specimens. 2015. PLoS Negl Trop Dis. 2015;25:9(9). doi: 10.1371/journal.pntd.0004100 26406240 PMC4583371

[51] Hirayama T, Mizuno Y, Takeshita N, Kotaki A, Tajima S, Omatsu T, et al. Detection of Dengue Virus Genome in Urine by Real-Time Reverse Transcriptase PCR: a Laboratory Diagnostic Method Useful after disappearance of the genome un serum. J Clin Microbiol. 2012;50(6):2047–52. 22442323 10.1128/JCM.06557-11 PMC3372171

[52] Barzon L, Pacenti M, Franchin E, Pagni S, Martello T, Cattai M, et al. Excretion of West Nile Virus in Urine During Acute Infection. J Infect Dis. 2013;208:1086–92. doi: 10.1093/infdis/jit290 23821721

[53] Gardner J, Rudd PA, Prow NA, Belarbi E, Roques P, Larcher T, et al. Infectious Chikungunya Virus in the Saliva of Mice, Monkeys and Humans. PLoS One. 2015;10(10). doi: 10.1371/journal.pone.0139481 26447467 PMC4598147

