# Supplementary material for: Prevalence of arboviruses and other infectious causes of skin rash in patients treated at a tertiary health unit in the Brazilian Amazon
Source: PLoS Negl Trop Dis. 2022 Oct 13;16(10):e0010727. doi: 10.1371/journal.pntd.0010727 (PMC9560595; doi:10.1371/journal.pntd.0010727)
Supplement: S1 Table — aMean ± Standard deviation. bUnadjusted p-value. cMann-Whitney test. dFisher exact test. eMinimum value: 1 day of symptom in the general population group, Zika positive group and negative group for all rash-causing infectious diseases tested in this study. Maximum value: 17 days of symptoms in the general population group, 16 days in the Zika positive group and 17 days in the negative group for all diseases tested. (DOCX) [file pntd.0010727.s001.docx]

| **Characteristics** | | **Zika positive (N=222)** | **Negative for all infections tested (N=86)** |  |
| --- | --- | --- | --- | --- |
|  |  | **N (%) or Mean ± SD^a^** | **N (%) or Mean ± SD** | ***p*-value^b^** |
| **Age (years)^c^** | | 38.4 ± 12.2 | 34.3 ± 12.2 | 0.0031 |
| **Age^d^** | 18 to 40 | 130 (58.6) | 61 (77.2) | 0.001 |
|  | 41 to 59 | 81 (36.5) | 14 (17.7) |  |
|  | ≥ 60 | 11 (4.9) | 4 (5.1) |  |
| **Gender^d^** | Male | 68 (30.6) | 29 (33.7) | 0.600 |
|  | Female | 154 (69.4) | 57 (66.3) |  |
| **Race^d^** | White | 29 (13.1) | 8 (9.3) | 0.652 |
|  | Black | 3 (1.3) | 0 |  |
|  | Brown | 186 (83.8) | 78 (90.7) |  |
|  | Indigenous | 1 (0.4) | 0 |  |
|  | Other | 3 (1.4) | 0 |  |
| **Days since onset of symptoms^ce^** | | 3.8 ± 2.3 | 4.1 ± 2.9 | 0.6361 |
| **Days since onset of symptoms^d^** | 0 to 2 | 66 (29.7) | 33 (38.4) | 0,015 |
|  | 3 to 5 | 115 (51.8) | 28 (32.5) |  |
|  | 6 to 8 | 36 (16.2) | 22 (25.6) |  |
|  | ≥ 9 | 5 (2.3) | 3 (3.5) |  |
| **Signs and symptoms^f^** | Maculopapular rash | 203 (91.4) | 75 (87.2) | 0.261 |
|  | Pruritus | 203 (91.4) | 67 (77.9) | 0.001 |
|  | Fever | 117 (52.7) | 35 (40.7) | 0.059 |
|  | Edema | 150 (67.6) | 46 (53.5) | 0.021 |
|  | Arthralgia | 167 (75.2) | 54 (62.8) | 0.030 |
|  | Conjunctival hyperemia | 146 (65.8) | 30 (34.9) | <0.001 |
