# Supplementary material for: Prevalence of arboviruses and other infectious causes of skin rash in patients treated at a tertiary health unit in the Brazilian Amazon
Source: PLoS Negl Trop Dis. 2022 Oct 13;16(10):e0010727. doi: 10.1371/journal.pntd.0010727 (PMC9560595; doi:10.1371/journal.pntd.0010727)
Supplement: S2 Table — aMean ± standard deviation. (DOCX) [file pntd.0010727.s002.docx]

| **Characteristics** | | **DENV (N=3)** | **CHIKV (N=1)** | **Parvov (N=3)** | **Measles (N=10)** | **HIV (N=1)** | **Syphilis (N=2)** | **Total (N=20)** |
| --- | --- | --- | --- | --- | --- | --- | --- | --- |
|  |  | **n (%) or Mean ± SD^a^** | **n (%) or Mean ± SD** | **n (%) or Mean ± SD** | **n (%) or Mean ± SD** | **n (%) or Mean ± SD** | **n (%) or Mean ± SD** | **n (%) or Mean ± SD** |
| **Age (years)** |  | 26.6 ± 5.77 | 25 | 30.33 ± 11.06 | 25.4 ± 6.04 | 41 | 28 (7.07) | 27.35 ± 7.06 |
|  | 18 to 40 | 3 (100.0) | 1 (100.0) | 2 (66.7) | 10 (100.0) | 0 | 2 (100.0) | 18 (90.0) |
|  | 41 to 59 | 0 | 0 | 1 (33.3) | 0 | 1 (100.0) | 0 | 2 (10.0) |
|  | ≥ 60 | 0 | 0 | 0 | 0 | 0 | 0 | 0 |
| **Gender** | Male | 2 (66.7) | 0 | 0 | 8 (80.0) | 0 | 1 (50.0) | 11 (55.0) |
|  | Female | 1 (33.3) | 1 (100.0) | 3 (100.0) | 2 (20.0) | 1 (100.0) | 1 (50.0) | 9 (45.0) |
| **Race** | White | 0 | 0 | 1 (33.3) | 0 | 0 | 1 (50.0) | 2 (10.0) |
|  | Black | 0 | 0 | 0 | 0 | 0 | 0 | 0 |
|  | Brown | 3 (100.0) | 1 (100.0) | 2 (66.7) | 10 (100.0) | 1 (100.0) | 1 (50.0) | 18 (90.0) |
|  | Indigenous | 0 | 0 | 0 | 0 | 0 | 0 | 0 |
|  | Other | 0 | 0 | 0 | 0 | 0 | 0 | 0 |
| **Days since onset of symptoms** | | 2 ± 0 | 2 | 5.66 ± 2.30 | 4.3 ± 2.45 | 6 | 3 ± 2.82 | 4.4 ± 2.13 |
|  | 0 to 2 | 0 | 0 | 0 | 2 (20.0) | 0 | 1 (50.0) | 3 (15.0) |
|  | 3 to 5 | 3 (100.0) | 1 (100.0) | 1 (33.3) | 6 (60.0) | 0 | 1 (50.0) | 12 (60.0) |
|  | 6 to 8 | 0 | 0 | 2 (66.7) | 1 (10.0) | 1 (100.0) | 0 | 4 (20.0) |
|  | ≥ 9 | 0 | 0 | 0 | 1 (10.0) | 0 | 0 | 1 (5.0) |
| **Signs and symptoms** | Maculopapular rash | 3 (100.0) | 1(100.0) | 3 (100.) | 10 (100.0) | 1 (100.0) | 1 (50.0) | 19 (95.0) |
|  | Pruritus | 3 (100.0) | 1(100.0) | 3 (100.0) | 8 (80.0) | 1 (100.0) | 1 (50.0) | 17 (85.0) |
|  | Fever | 3 (100.0) | 1 (100.0) | 1 (33.3) | 6 (60.0) | 1 (100.0) | 1 (50.0) | 13 (65.0) |
|  | Edema | 1 (33.3) | 1 (100.0) | 3 (100.0) | 8 (80.0) | 0 | 1 (50.0) | 14 (70.0) |
|  | Arthralgia | 2 (66.7) | 1 (100.0) | 2 (66.7) | 5 (50.0) | 1 (100.0) | 2 (100.0) | 13 (65.0) |
|  | Conjunctival hyperemia | 1 (33.3) | 1 (100.0) | 3 (100.0) | 7 (70.0) | 1 (100.0) | 1 (50.0) | 10 (50.0) |
