# Supplementary material for: Prevalence of arboviruses and other infectious causes of skin rash in patients treated at a tertiary health unit in the Brazilian Amazon
Source: PLoS Negl Trop Dis. 2022 Oct 13;16(10):e0010727. doi: 10.1371/journal.pntd.0010727 (PMC9560595; doi:10.1371/journal.pntd.0010727)
Supplement: S3 Table — EBV: Epstein-Barr; CHIKV: Chikungunya virus; CMV: Cytomegalovirus; HIV: Human Immunodeficiency Virus; ZIKV: Zika virus. aMean ± standard deviation. (DOCX) [file pntd.0010727.s003.docx]

| **Characteristics** | | **ZIKV + HIV (N=1)** | **ZIKV + Measles (N=1)** | **ZIKV + Parvovirus B19 (N=1)** | **ZIKV + EBV (N=1)** | **EBV + Parvovirus B19 (N=1)** | **CMV + Parvovirus B19 (N=2)** | **CMV + Syphilis (N=1)** | **ZIKV + EBV + Parvovirus B19 (N=1)** | **CMV + EBV + Parvovirus B19 (N=3)** | **Total (N=12)** |
| --- | --- | --- | --- | --- | --- | --- | --- | --- | --- | --- | --- |
|  |  | **n (%) or Mean ± SD1** | **n (%) or Mean ± SD** | **n (%) or Mean ± SD** | **n (%) or Mean ± SD** | **n (%) or Mean ± SD** | **n (%) or Mean ± SD** | **n (%) or Mean ± SD** | **n (%) or Mean ± SD** | **n (%) or Mean ± SD** | **n (%) or Mean ± SD** |
| **Age (years)** |  | 33 | 21 | 20 | 45 | 42 | 30 ± 5.7 | 38 | 30 | 33 ± 5.6 | 32.33 ± 7.9 |
|  | 18 to 40 | 1 (100.0) | 1 (100.0) | 1 (100.0) | 0 | 0 | 2 (100.0) | 1 (100.0) | 1 (100.0) | 3 (100.0) | 10 (83.3) |
|  | 41 to 59 | 0 | 0 | 0 | 1 (100.0) | 1 (100.0) | 0 | 0 | 0 | 0 | 2 (16.7) |
|  | ≥ 60 | 0 | 0 | 0 | 0 | 0 | 0 | 0 | 0 | 0 | 0 |
| **Gender** | Male | 1 (100.0) | 1 (100.0) | 1 (100.0) | 0 | 0 | 0 | 1 (100.0) | 0 | 0 | 4 (33.3) |
|  | Female | 0 | 0 | 0 | 1 (100.0) | 1 (100.0) | 2 (100.0) | 0 | 1 (100.0) | 3 (100.0) | 8 (66.7) |
| **Race** | White | 0 | 0 | 0 | 0 | 0 | 0 | 0 | 0 | 1 (33.3) | 1 (8.3) |
|  | Black | 0 | 0 | 0 | 0 | 0 | 0 | 0 | 0 | 0 | 0 |
|  | Brown | 1 (100.0) | 1 (100.0) | 1 (100.0) | 1 (100.0) | 1 (100.0) | 2 (100.0) | 1 (100.0) | 1 (100.0) | 2 (66.7) | 11 (91.7) |
|  | Indigenous | 0 | 0 | 0 | 0 | 0 | 0 | 0 | 0 | 0 | 0 |
|  | Other | 0 | 0 | 0 | 0 | 0 | 0 | 0 | 0 | 0 | 0 |
| **Days since onset of symptoms** | | 3 | 4 | 3 | 5 | 6 | 2.5 ± 2.1 | 6 | 5 | 6 ± 3.5 | 4.6 ± 2.2 |
|  | 0 to 2 | 0 | 0 | 0 | 0 | 0 | 1 (50.0) | 0 | 0 | 0 | 1 (8.3) |
|  | 3 to 5 | 1 (100.0) | 1 (100.0) | 1 (100.0) | 1 (100.0) | 0 | 1 (100.0) | 0 | 1 (100.0) | 2 (66.7) | 8 (66.7) |
|  | 6 to 8 | 0 | 0 | 0 | 0 | 1 (100.0) | 0 | 1 (100.0) | 0 | 0 | 2 (16.7) |
|  | ≥ 9 | 0 | 0 | 0 | 0 | 0 | 0 | 0 | 0 | 1 (33.3) | 1 (8.3) |
| **Signs and symptoms** | Maculopapular rash | 1 (100.0) | 1 (100.0) | 1 (100.0) | 1 (100.0) | 1 (100.0) | 2 (100.0) | 1 (100.0) | 1 (100.0) | 2 (66.7) | 11 (91.7) |
|  | Pruritus | 1 (100.0) | 1 (100.0) | 0 | 1 (100.0) | 1 (100.0) | 2 (100.0) | 1 (100.0) | 1 (100.0) | 3 (100.0) | 11 (91.7) |
|  | Fever | 1 (100.0) | 0 | 1 (100.0) | 0 | 0 | 1 (50.0) | 1 (100.0) | 1 (100.0) | 2 (66.7) | 7 (58.3) |
|  | Edema | 0 | 0 | 0 | 1 (100.0) | 1 (100.0) | 1 (50.0) | 0 | 0 | 2 (66.7) | 5 (41.7) |
|  | Arthralgia | 1 (100.0) | 0 | 0 | 1 (100.0) | 1 (100.0) | 2 (100.0) | 1 (100.0) | 0 | 3 (100.0) | 9 (75.0) |
|  | Conjunctival hyperemia | 0 | 1 (100.0) | 1 (100.0) | 0 | 0 | 1 (50.0) | 1 (100.0) | 0 | 1 (33.3) | 5 (41.7) |
